# The 2019–2020 Rise in Lake Victoria Monitored from Space: Exploiting the State-of-the-Art GRACE-FO and the Newly Released ERA-5 Reanalysis Products

**DOI:** 10.3390/s21134304

**Published:** 2021-06-23

**Authors:** Mehdi Khaki, Joseph Awange

**Affiliations:** 1 School of Engineering, University of Newcastle, Callaghan 2308, Australia; Mehdi.Khaki@Newcastle.edu.au; 2 School of Earth and Planetary Sciences, Spatial Sciences, Curtin University, Perth 6102, Australia

**Keywords:** Lake Victoria, data assimilation, satellite radar altimetry, GRACE follow-on, water storage changes, land surface modelling

## Abstract

During the period 2019–2020, Lake Victoria water levels rose at an alarming rate that has caused various problems in the region. The influence of this phenomena on surface and subsurface water resources has not yet been investigated, largely due to lack of enough in situ measurements compounded by the spatial coverage of the lake’s basin, incomplete/inconsistent hydrometeorological data, and unavailable governmental data. Within the framework of joint data assimilation into a land surface model from multi-mission satellite remote sensing, this study employs the state-of-art Gravity Recovery and Climate Experiment follow-on (GRACE-FO) time-variable terrestrial water storage (TWS), newly released ERA-5 reanalysis, and satellite radar altimetry products to understand the cause of the rise of Lake Victoria on the one hand, and the associated impacts of the rise on the total water storage compartments (surface and groundwater) triggered by the extreme climatic event on the other hand. In addition, the study investigates the impacts of large-scale ocean–atmosphere indices on the water storage changes. The results indicate a considerable increase in water storage over the past two years, with multiple subsequent positive trends mainly induced by the Indian Ocean Dipole (IOD). Significant storage increase is also quantified in various water components such as surface water and water discharge, where the results show the lake’s water level rose by ∼1.4 m, leading to approximately 1750 gigatonne volume increase. Multiple positive trends are observed in the past two years in the lake’s water storage increase with two major events in April–May 2019 and December 2019–January 2020, with the rainfall occurring during the short rainy season of September to November (SON) having had a dominant effect on the lake’s rise.

## 1. Introduction

Lake Victoria, the world’s second largest freshwater lake that supports over 42 million people within its basin (e.g., [1]) has recently undergone unprecedented changes caused by multiple factors such as heavy rains, human activities, environmental degradation, and urbanization, which have led to a significant water level rise (e.g., [2,3]). In the past two years (2019–2020), the lake’s water level increased remarkably to an all-time record that resulted in floods, impacts on drinking water and sanitation systems, increased water-related diseases, and impacts on hydropower infrastructures [4]. In addition to providing water resources, the lake supports the region’s agriculture, fisheries, and hydropower, and is also known to modulate the regional climate (see, e.g., [1,5]). These vital roles played by the lake suggest the fact that its short to long-term changes can significantly impact people’s livelihood in the region and beyond (see, e.g., [2]).

A study by [6] showed that under various climate projection, the Lake Victoria basin will experience a substantial increase in mean annual minimum temperature, which will lead to high river discharge variability. They concluded that this makes it necessary to better coordinate transboundary river management in the future. Ref. [7] also pointed out the impact of climate change on the lake’s region and reported more than 1° temperature rise since 1927. The sensitivity of Lake Victoria to the climatic impacts can be due to the fact that precipitation and evaporation play the most important role in the lake’s water storage [2,8,9,10]. Any considerable changes in these water components can then have a significant impact on the lake region by causing droughts or floods. Ref. [11] discussed the necessity of monitoring the lake water changes particularly caused by extreme climatic events to better understand their effect on water quality and water circulation for climate adaptation and the region’s development. These notwithstanding, the impacts of the 2019–2020 extreme climate event on various water components (surface and underground) has not yet been investigated.

The primary objective of this work, therefore, is to study the recent water storage changes over Lake Victoria due to its significant impact on the region (see, e.g., [12]). To this end, various tools including a land surface hydrological model and satellite remote sensing data are used. These are particularly useful in the region due to the lack of widely spread in situ hydrometeorological stations on the one hand, and difficulties in accessing these data from the limited exiting stations where available due to governmental bureaucracies on the other hand [1,4]. Besides, where such hydrometeorological data are accessible, they are often either incomplete and/or inconsistent thereby difficult to be used. One way to address this data shortage is to use land surface models or rely on satellite remote sensing products (e.g., [13,14,15]). These alternatives offer high-resolution data over an extended period in many parts of the world including the Lake Victoria region (e.g., [16,17,18]).

The models can generate various water component estimates such as groundwater, surface water, and soil moisture and have been applied for monitoring water storage changes in the region (see, e.g., [6,19,20,21]). Ref. [22] used a hydrological model to estimate the water balance of Lake Victoria and to analyse evaporation, precipitation, and discharge changes (see also [23]). Ref. [24] studied the lake’s water budget and the impacts of climate change using models. Lake Victoria water quality and ecosystem change monitoring has also been done based on developed models (e.g., [25,26]). These models, however, can be subject to errors due to imperfect modelling, inaccurate model parameters, and error in forcing data and model inputs [27,28]. Merging satellite remote sensing data with the land surface models can address these issues by correcting model simulations based on direct satellite measurements. This is especially of interest knowing different satellite platforms can deliver a range of different observations, e.g., water level data and terrestrial water storage (TWS).

This study employs the state-of-the-art GRACE Follow-on (GRACE-FO) and the newly released ERA-5 reanalysis products and model data within the data assimilation scheme to understand (i) the cause of the 2019–2020 increase in the Lake Victoria water level, and (ii) the impacts of the lake level rise on its total water storage compartments (surface and groundwater). Data assimilation allows for an integration of independent datasets (e.g., satellite remote sensing) into the models, hence improving their estimates. Here, for the first time, we apply TWS from the GRACE-FO mission and surface water storage derived from multi-mission satellite radar altimetry simultaneously in the assimilation process. This is carried out to improve the model estimates of various water compartments over the past two years that include the current rise of the lake. GRACE TWS data assimilation has been previously used in hydrology and proves to successfully enhance the model performance (see, e.g., [29,30,31]). Assimilation of altimetry-derived water storage has also been applied recently in [21] and effectively improved model surface water storage estimates. This is the first multivariate data assimilation over Lake Victoria, particularly designed to study the 2019–2020 hydroclimatic event within the region.

From the information derived from the implementation of the above assimilation approach, output of water storage changes as TWS (downgraded into higher spatiotemporal resolution from GRACE), groundwater, and surface water can be separately analysed. The impacts of precipitation and also large-scale ocean-atmosphere indices including El Niño Southern Oscillation (ENSO), Indian Ocean Dipole (IOD) and North Atlantic Oscillation (NAO) on water storage changes are also investigated to identify the main trigger of the significant water changes in the Lake Victoria region during the 2019–2020 period. Climate variabilities impact, especially caused by IOD phenomena on the Nile basin and lake Victoria basins have been previously reported over various time periods (see, e.g., [4]). This demonstrates the ever-increasing influence of climate change on the Lake Victoria region that is further investigated in the present study (Section 4). In what follows, model and data are presented in Section 3.1. The data assimilation methodology is presented in Section 3.2 and the results discussed in Section 4 before concluding the study in Section 6.

## 2. Lake Victoria

**Figure 1:**
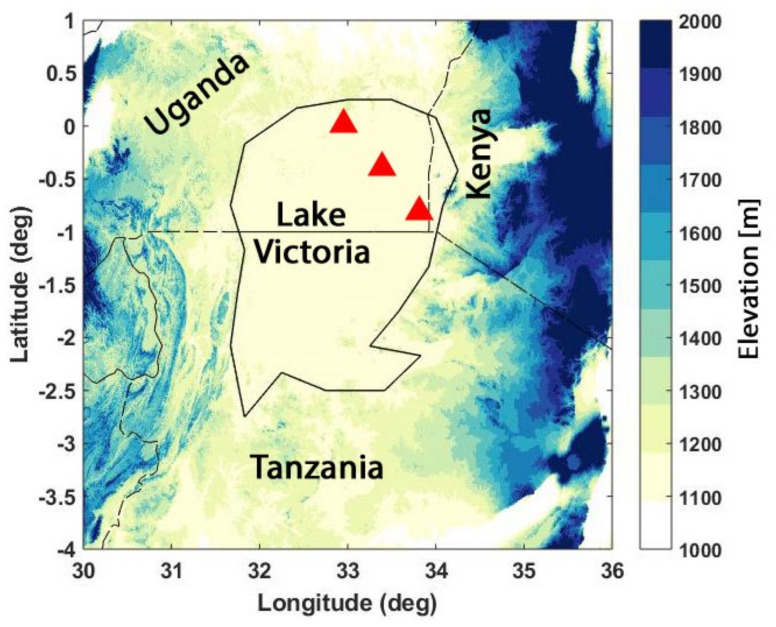
The Lake Victoria boundary (black) with the borders of Kenya, Uganda and Tanzania presented on the region’s digital elevation map. The location of virtual stations for the altimetry water level data are presented in red triangles.

Lake Victoria is the largest lake (approximate area of 69,295 ${\mathrm{km}}^{2}$; Refs. [1,4]) in Africa and the second-largest freshwater lake in the world [1]. It is located within the Lake Victoria basin (LVB) in the southern (geographically) part of the Nile River basin (see, e.g., [4]). The lake, which is located upstream of the Nile, draws 20% of its water from inflow rivers such as Kagera, Gurumeti, and Simiyu and 80% from precipitation [12]. Except for the northern part of Lake Victoria Basin (LVB), it is surrounded by mountains (Figure 1). The lake has been affected by both human impacts and climate change over the years [2]. Poor agricultural practices and deforestation have increased the lake’s sedimentation and influx streamflow [12,32]. Climate change has also significantly influenced the lake as it is very sensitive to rainfall and evaporation [5]. The lake’s temperature has risen by 0.5°C since 1960 and its hydrological cycle has changed to balance between temperature and rainfall (see, e.g., [33]). It is reported that extreme climatic events (e.g., 1997 El Niño and 2007 ENSO rainfall effect) have increased in frequency and strength [34,35]. These emphasise the importance of constant and accurate water storage monitoring of the lake due to its vulnerability from extreme and/or frequent climatic influences.

## 3. Materials and Methods

### 3.1. Model and Data

#### 3.1.1. Land Surface Model

Global hydrological models can be employed to study water storage components over the Lake Victoria region given the lack of in situ measurements and local models. The Commonwealth Scientific and Industrial Research Organisation (CSIRO) model of W3RA (World-Wide Water Resources Assessment) is used here. The model performs on a grid base and simulates interactions between water and energy, i.e., through the distribution of precipitation between various components of evaporation, net precipitation, runoffs, and infiltration [36]. The model outputs include (but are not limited to) water stored at different depths (top layer, shallow- and deep-root zones), surface water, groundwater, and snow water. Various parameters at every grid point are used for the modelling objectives such as effective soil parameters, water holding capacity that relates to groundwater recession and greenness, as well as the saturated area to catchment characteristics. To run the model, the daily meteorological forcing datasets are derived from the ERA-5 reanalysis. ERA5, which replaced the ERA-Interim reanalysis, contains various atmospheric, land and oceanic climate datasets. The data covers the entire Earth’s surface up to a height of 80 km using 137 atmospheric levels. Here, we use daily precipitation, downwelling short-wave radiation, and maximum and minimum temperature at 0.1250° spatial resolution. For the data assimilation experiment, we focus on water storage compartments of groundwater, soil moisture at all layers, surface water, and snow water storage. These will be updated via data assimilation scheme (cf. Section 3.2.1).

#### 3.1.2. Satellite Remote Sensing

Two main satellite data products from GRACE and radar altimetry are used for the assimilation. The data (described below) are derived for the period of January 2019 to July 2020 over the LV region.

##### GRACE-FO TWS

The TWS data are derived from the GRACE-FO mission. Level 2 (L2) products up to degree and order 120 are acquired from the monthly ITSG-Grace2018 gravity field model [37]. The data, which are in form of spherical harmonics are preprocessed using a standard procedure that includes replacing low degree coefficients (i.e., C10, C11, S11, and C20) with accurate estimates [38,39]. They are then converted to TWS change at 1°×1° spatial resolution following [40]. To account for striping error and tackle the leakage effects, the Kernel Fourier Integration (KeFIn) filter [41] is applied. The method applies two consecutive steps to (1) mitigate the measurement noise and the aliasing of unmodelled mass variations, and (2) reduce the leakage using an efficient anisotropic kernel to isolate the signals in the region of interest (see details of data processing in [41]).

##### Satellite Radar Altimetry

The Sensor Geographic Data Records (SGDR) from Jason-3 mission is obtained from the National Oceanographic Data Center (NODC) to estimate water level data. The datasets are corrected for geophysical corrections including solid earth tide and pole tide [42]. The data with the 10-day repeat cycle contain 20 hz waveform values, which allows for the application of retracking process. Following [43], Extrema Retracking (ExtR) algorithm is applied to retrack the waveform measurements in order to tackle the altimetry data issues over the inland water bodies, which is caused by poor signal scattered over shallow waters (compared to oceans) as well as where land and water meet. The applied ExtR method comprises two steps including (1) eliminating outliers (data snooping procedure following [44]) and noisy waveforms from satellite observations, and (2) implementing retracking to derive the correct altimetry range measurements. The method has been successfully implemented and evaluated over the lakes (e.g., Lake Victoria) with promising performance against the existing approaches (e.g., [18,43]). The applied retracking method improves the range measurements and subsequently water level variations over lakes and inland waters. The generated water level at multiple points from the same satellite cycle over LV is used to address the hooking effects [45] by averaging the values of the retracked altimetry-based water levels.

#### 3.1.3. Precipitation and Climate Variability Data

Daily precipitation data are derived from Global Precipitation Measurement (GPM; [46]). The data is used to explore the relationship between precipitation and water storage changes estimated from the data assimilation approach. Moreover, to further investigate the impact of large-scale ocean–atmosphere indices, three climate indicators of El Niño Southern Oscillation (ENSO), Indian Ocean Dipole (IOD), and North Atlantic Oscillation (NAO) are used. The data are downloaded for the study period from Climate Prediction Center (CPC). The full list of datasets used is presented in Table 1.

### 3.2. Methods

#### 3.2.1. Data Assimilation

The application of data assimilation has been successfully proven when GRACE TWS (e.g., [29,30,31,48,49]) and altimetry-derived products (see, e.g., [21,50,51]) are used. The process improves model estimates according to the observations. Data assimilation has previously been used over the region (see, e.g., [21]) and found to be effective for enhancing W3RA water storage compartments hence applied here. In order to assimilate GRACE TWS and surface water storage from satellite altimetry into the W3RA model, ensemble Kalman filter (EnKF) is used. EnKF is based on sequential data assimilation scheme that performs in two successive operations at every assimilation cycle when new observation (e.g., from GRACE and/or satellite altimetry) is available. These two operations include (i) a forecast step, and (ii), an analysis step. In the forecast step, the model state vector at previous time step (t−1) containing water storage components of groundwater, soil moisture at all layers, surface water, and snow water storage advances in time to *t* using the dynamical model (M). This can be written as,

(1) $$x_{t}^{f,(i)}=M\left(x_{t-1}^{a,(i)}\right)+v^{\left(i\right)},\;i=1,\ldots ,n,$$

where ${\left\{x_{t}^{f,(i)}\right\}}_{i=1}^{n}$ is the forecast state at *t* and ${\left\{x_{t-1}^{a,(i)}\right\}}_{i=1}^{n}$ (*n* refers to ensemble number) is the analysis state at t−1. $v^{\left(i\right)}$ in Equation (1) represents Gaussian random sample with covariance matrix $Q_{t}$. The model dynamic M includes the underlying model equations that are used to transfer state vector one time step forward (t−1 to *t*). These equations in W3RA are based on a water balance system that simulates various water storage components separately for each grid based on incoming (e.g., precipitation) and outgoing (e.g., runoff) water and energy fluxes. Full details of the model’s description and equations can be found in [52].

Next, the forecast state is estimated according to the incoming observations ($y_{t}$) via an EnKF analysis step,

(2) $$\begin{array}{ccc} y_{t}^{f,(i)} & = & Hx_{t}^{f,(i)}+w^{\left(i\right)};\;i=1,\ldots ,n, \end{array}$$

(3) $$\begin{array}{ccc} x_{t}^{a,(i)} & = & x_{t}^{f,(i)}+\underset{K_{t}^{\left(i\right)}}{\underbrace{P_{x_{t}^{f}}H^{T}{\left[HP_{x_{t}^{f}}H^{T}+R_{t}\right]}^{-1}}}\left[y_{t}-y_{t}^{f,(i)}\right],\;i=1,\ldots ,n. \end{array}$$

H represents the observation transition matrix, which is used to translates model space into that of observation. This has two main blocks: (1) to aggregate all state components including groundwater, soil moisture at all layers, surface water, and snow water storage to be updated by GRACE TWS, and (2) to only update surface water storage with the altimetry-derived observations. $w^{\left(i\right)}$ is the observation Gaussian noise with observation error covariance $R_{t}$, which is assumed to be uncorrelated and linearly depend on observations at each grid point, i.e., $R_{t}={\left(\alpha .y_{t}\right)}^{2}$ with α=0.2 for GRACE (as suggested by [53]) and α=0.1 for altimetry data (e.g., [54]). In Equation (3), $K_{t}^{\left(i\right)}$ refers to Kalman gain and $P_{x_{t}^{f}}$, which is the state covariance matrix can be estimated from,

(4) $$\begin{array}{ccc} P_{x_{t}^{f}} & = & {\left(n-1\right)}^{-1}S_{x_{t}^{f}}S_{x_{t}^{f}}^{T}, \end{array}$$

where $S_{x_{t}^{f}}$ presents the state perturbation matrix. $S_{x_{t}^{f}}$ can be calculated by subtracting the ensemble mean from the ensemble members’ value. At this stage, the analysis state and its corresponding covariance matrix are updated and will be integrated with the model to the next time step. This process will be repeated at every assimilation cycle.

#### 3.2.2. Experimental Design

The data assimilation process begins with the ensemble generation. To this end, the model is run for 2018 using n=30 (30 ensemble) different scenarios imposed by meteorological forcing fields perturbation as ×N(0,0.3) (i.e., Gaussian with zero mean and 0.3 variance) for precipitation, +N(0,50) for shortwave radiation, and +N(0,2) for temperature, which lead to ensemble of m=30 at the beginning of the study period (January 2019). The state vector to be updated during data assimilation includes soil moisture, snow and surface storage, and groundwater. The observation vector includes altimetry derived surface water storage and GRACE TWS observations. To obtain surface water storage from the altimetry products, we follow [55] to derive grid point water levels from the established virtual station on altimetry footprints using a bilinear interpolation method. These are then used to estimate surface water storage changes between two consecutive data acquisitions (see details in [45,55]). Ref. [21] reported that the estimated surface water storage can successfully improve the model, thus, a similar approach is applied here. Furthermore, Cumulative Distribution Function (CDF) is applied to remove the bias between the two observation sets and the counterpart model estimates.

**Figure 2:**
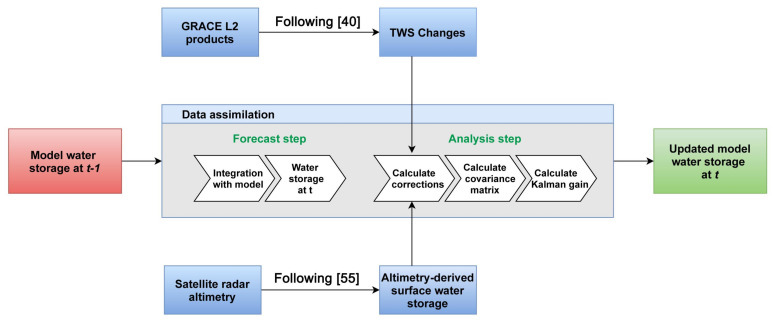
The implemented data assimilation scheme to integrate GRACE TWS and altimetry-derived surface water storage with the model.

At every assimilation cycle, state variables (at ∼12 km resolution) are converted into the observation space (in 1°×1° resolution) using the observational operator ($H_{t}$). Therefore, the model surface water storage is then updated by the altimetry-derived surface data and aggregated model’s state variables are updated by the GRACE-FO TWS data. EnKF, which is applied to perform these state updates, is sensitive to the number of ensemble and better results are expected with a higher ensemble number [56]. However, this will significantly add to computation needs. Two standard tuning techniques are usually applied to tackle this issue; i.e., ensemble inflation and localization. Here, ensemble inflation with the coefficient of 1.2 (suggested by the literature, e.g., [56]) is applied to avoid filter divergent. Moreover, Local Analysis (LA) scheme with the radius of 2° (e.g., suggested by [57]) is used to spatially limit the update process and improve the estimates. The implemented data assimilation process and its associated steps are summarised in Figure 2.

#### 3.2.3. Correlation Analysis

Correlation analysis is used throughout the study to compare the results with the data. To this end, the data assimilation results are temporally rescaled to that of data, e.g., monthly for GRACE using the spline interpolation. Note that precipitation data is provided on a daily scale same as the model outputs. To assess the significance of the correlation results, *p*-values of the estimates are calculated based on t-distribution at 95% confidence level.

## 4. Results

### 4.1. Assimilation Results

**Figure 3:**
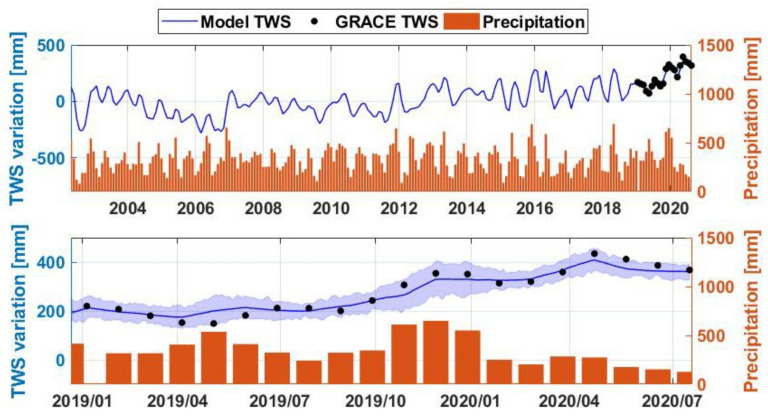
(**Top**) Average TWS change and precipitation time series over the Lake Victoria region during the period 2002–2020; (**Bottom**) Time series are represented for the period 2019–2020 with the assimilation uncertainty in shaded blue. More rains and subsequent lake level increase occur during the short rainy season of September–October–November (SON) compared to the long rainy season of March–April–May (MAM).

Analysing water storage changes within the Lake Victoria region is presented, where the average time series of TWS changes from data assimilation is compared with the precipitation time series in Figure 3. The gridded TWS changes and precipitation products are spatially averaged over the lake to produce the time series. The time series are extended to the period of 2002 to 2020 to better investigate the recent changes. Significant water storage increase can be seen during 2019–2020. The positive trend for this period can be seen in both the GRACE-FO and the assimilation results. Note that the data assimilation covers only the period with the GRACE data. A lower trend rate can also be seen after 2016, which is exacerbated from 2019 leading to a remarkable storage increase in early 2020. This can be better seen in Figure 3 bottom panel. The presented data assimilation uncertainties calculated using the model ensemble also show the overall trend and confirms the TWS increase compared to the previous periods. It can also be seen from Figure 3 that TWS changes largely follow the precipitation pattern, which indicates the great impact of rainfall on the lake’s water storage. The applied cross-correlation between the TWS changes and precipitation time series indicates the maximum correlation coefficient of 0.86 with a lag time of two months. Two significant positive trends are evident from the figure (particularly for precipitation), i.e., in April–May 2019 (i.e., long rainy season of March–April–May MAM) followed by a larger anomaly in December 2019–January 2020. During 2020, however, due to the precipitation decline, the TWS change trend becomes negative after April 2020.

From Figure 3, it can be seen that the assimilation results closely match the GRACE-FO changes as expected due to the applied data assimilation process. The application of data assimilation increases the correlation between the model and GRACE observations from 0.76 to 0.94. To better explore the impact of data assimilation for improving the model estimates, in the lack of in situ measurements, we compare the TWS results to those of three widely used land surface models including 0.50° outputs (for the assimilation period) from the Community Land Model (CLM version 4.5; [58]), PCR-GLOBWB (version 2; [59]), and WaterGAP (version 2; [60]). The average correlation values between the TWS estimates and CLM4.5 TWS, PCR-GLOBWB, and WaterGAP2 are 0.92, 0.85, and 0.88, respectively. The three outputs are also merged following [61] using Triple Collocation Analysis (TCA) and then used to evaluate the TWS estimates. Results indicate an average reduction of 9.29 mm Root Mean Square Error (RMSE) from 23.66 mm without data assimilation. This shows the ability of data assimilation to reduce the model errors.

**Figure 4:**
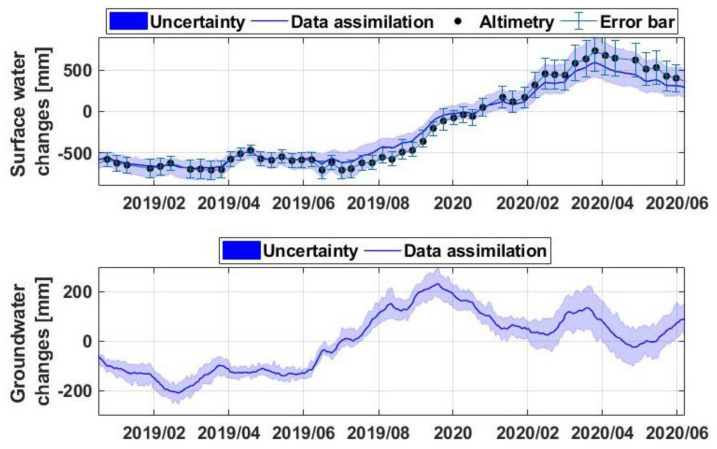
Average surface water (**top**) and groundwater (**bottom**) changes over LV. The associated uncertainties derived from the model ensemble are also displayed for both water components. Altimetry results with their errors are presented in the top panel against the data assimilation surface water storage estimates.

Surface water storage and water fluctuations in Lake Victoria are further analysed using the assimilation results and water level changes from satellite radar altimetry. Figure 4 shows the average surface water storage changes along with the estimated uncertainties over Lake Victoria. Surface water measurements from altimetry are also shown in the figure, which indicate a good agreement between the two time series. It is found that the average correlation between the surface water storage and altimetry data increased from 0.52 (without data assimilation) to 0.75 (with data assimilation) due to the assimilation impact. This shows the positive impact of data assimilation in reducing the discrepancy between the model and altimetry observations. The two previously discussed positive anomalies can also be seen in the time series. The second surge is similarly more pronounced leading to a lake’s water level record in mid-2020 (1.387 m above the similar time in 2019). This is mainly caused by the above-average precipitation that occurred during 2019 in both the long (MAM) and short (SON) rainy seasons that resulted in a significant surface water rise in Figure 4. Based on the results, the lake’s water volume increased by approximately 1750 gigatonne over the study period. A similar finding is achieved using the altimetry water level data and the region’s digital elevation model (DEM from [62]) to calculate the lake’s volume.

This remarkable storage increase over a relatively short period had disastrous impacts on the surrounding countries. Nevertheless, with the decrease in the amount of precipitation with an overall negative trend in 2020 and increased evaporation, surface water declined by July 2020 similar to TWS changes in Figure 3. Significantly larger water increase in April–May 2019 and early 2020 from the surface storage, water level and TWS change time series compared to the precipitation (with relatively smaller peaks) can be explained by accumulated precipitated water across 2019 as well as the impact of inflow water from the rivers discharging into the lake. This can also be seen in the groundwater time series. The positive trend is evident in late 2019 to early 2020 in the average groundwater estimates. The presented groundwater uncertainty also shows that the trend, especially between mid-2019 to early 2020 is considerable. This positive trend is then followed by a negative trend as a result of the rainfall decline through 2020.

### 4.2. Hydroclimate Variability

**Figure 5:**
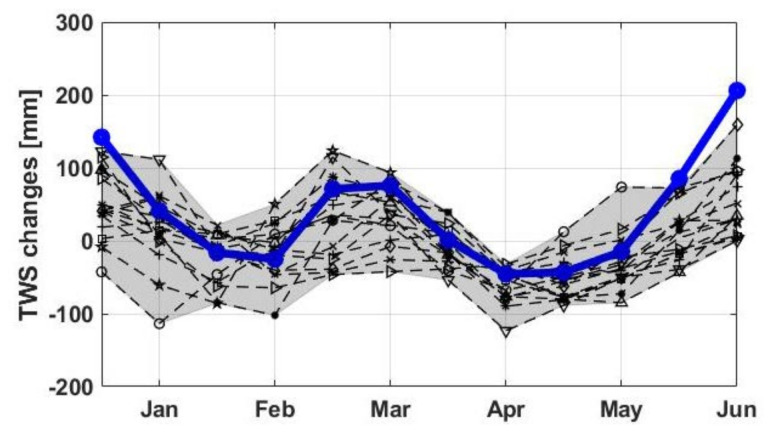
Average water storage changes for each month from 2002 to 2018; each year is represented by a black dashed line and different symbols (shaded grey area shows the range of variations). The blue line indicates water storage change for the 2019–2020 period, showing a higher than normal TWS increase compared with the previous period.

**Figure 6:**
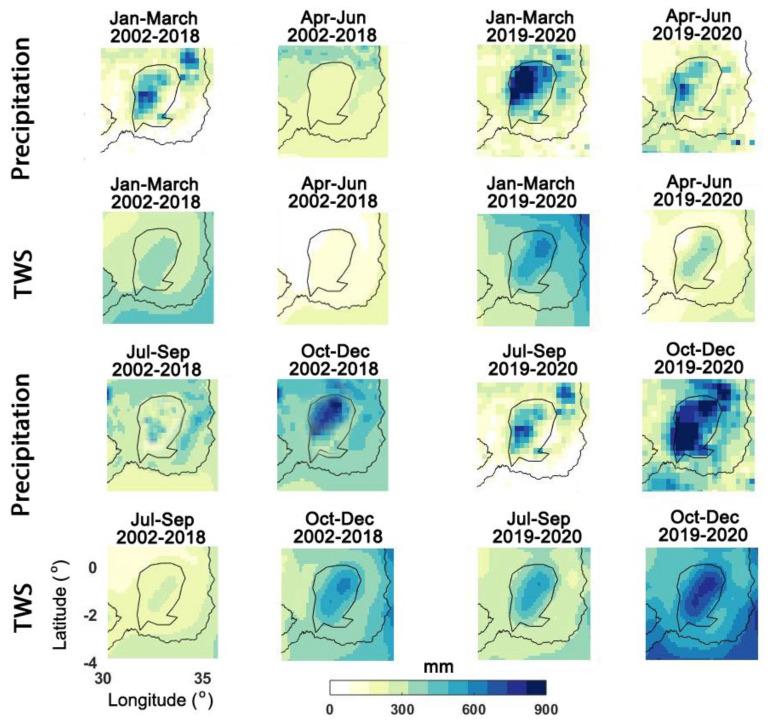
Average TWS and precipitation maps for various periods within the LV region.

To further explore the 2019–2020 water storage increase over the Lake Victoria region, seasonal TWS changes are analysed. To this end, average TWS changes for every month for the period of 2002 to 2018 (grey graphs in Figure 5) are compared with those of 2019 to 2020 (blue graph in Figure 5). Note that every grey graph represents one year of results while blue indicates the average over during the 2019–2020 period. Higher than average TWS results for the wet season, i.e., September to November is evident for the last two years against the period before that. This is clearer for the months December and January showing the impact of TWS increase, much larger than previous years. The higher-than-usual TWS during the dry season, i.e., June to August, again indicates the substantial water content available within the region for 2019–2020. The described changes can also be seen in Figure 6, where average TWS and precipitation for different periods are displayed.

**Figure 7:**
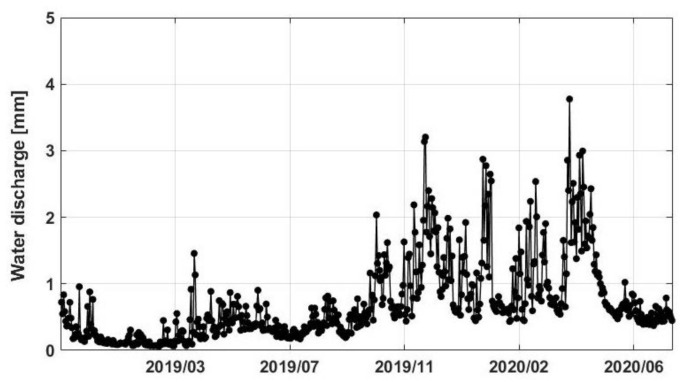
Average water discharge from the assimilation results over LV.

**Figure 8:**
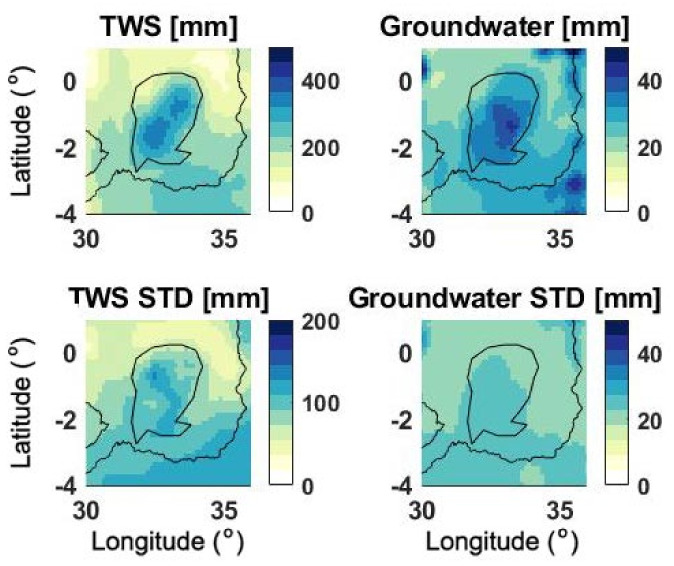
Temporally averaged TWS and groundwater values, both calculated for the period of January 2019 to July 2020 from data assimilation. The corresponding STD maps calculated from data assimilation are displayed in the bottom row.

**Figure 9:**
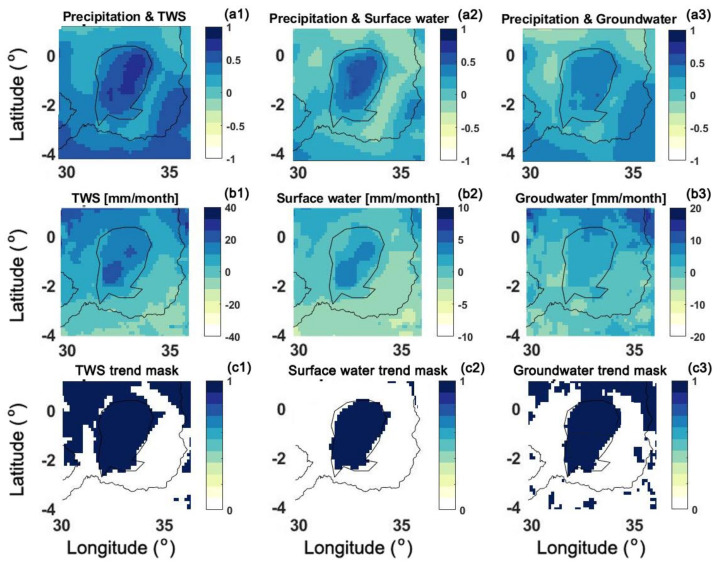
Correlation between precipitation and TWS (**a1**), surface water (**a2**), and groundwater (**a3**) for the period of January 2019 to July 2020. TWS, surface water storage, and groundwater trends estimated from data assimilation results are presented in (**b1**), (**b2**), and (**b3**), respectively. The corresponding trend mask are plotted in (**c1**), (**c2**), and (**c3**), respectively, which show the grid points with significant trend values.

**Figure 10:**
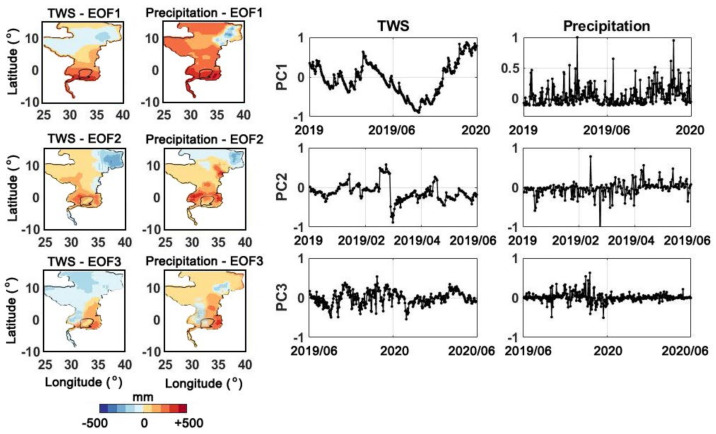
The first three components of PCA applied on TWS and precipitation time series.

Figure 6 shows TWS and precipitation changes separately for the period of 2002–2018, and 2019 to 2020. This is done to better focus on the changes in the period 2019–2020 against the previous years. Each grid point in the maps represents the average values in the corresponding period. The TWS average maps follow the rainfall pattern to a large extent. It can be seen that both precipitation and TWS maps demonstrate considerably higher water content after 2019. This is clearer for January–March and particularly for October–December. Overall, similar to the precipitation, water storage increase in 2019 can be seen with the largest amount of TWS occurring in October–December 2019. This is also indicated in Figure 3, Figure 4, Figure 5, Figure 6, Figure 7, Figure 8, Figure 9 and Figure 10 as TWS change time series show a similar storage rise over the same period. Another positive anomaly over the lake area can be seen for July–September 2019–2020 for both precipitation and TWS maps. This, again, confirms the previous results, in which water storage increase was observed particularly in April 2019. Such positive precipitation and TWS, which also exist to a less degree in April–June cannot be seen in the previous years and over the dry season (June to August). To better investigate these changes, principal component analysis (PCA [63]) is applied to gridded TWS changes and precipitation time series (see Section 4.4).

To better investigate the discharge influence of the rivers into the lake, the data assimilation results of water discharge is used. The spatially averaged discharge estimated over LV for the study period is depicted in Figure 7. As can be seen, the graph presents a significant increase in December 2019–January 2020 followed by another strong anomaly in March 2020. The former positive discharge change could also be seen in Figure 3 and Figure 6. Similarly, an increase can be observed for the period of April–May 2019. The March 2020 discharge increase rate is more pronounced than TWS change and water level time series over the same period. This could be due to the impact of changes in inflow water discharge from upstream sources such as Kagera, Gurumeti, and Simiyu as a result of increased precipitation (as can also be seen in Figure 3). This stream flow increase, again, is followed by a negative trend up to mid-2020, similar to those of TWS change and water level time series.

Next, spatial variations of water components are considered. Figure 8 shows spatial variations of TWS and groundwater. Every grid point in the figure corresponds to the temporal average of the TWS and groundwater from data assimilation. The corresponding standard deviations (STD) at each grid point for the same period are also calculated and shown in Figure 8. Increased water storage can be seen in both water components as a result of (i) excess precipitation, and (ii) streamflow growth as shown previously. This was expected due to a large amount of rainfall particularly in 2019. Larger STD values are also found for both TWS and groundwater within the lake area, which indicate the increased variability of these water components. This is attributed to the TWS and groundwater changes in the region over the period 2019–2020 as also observed in Figure 3 and Figure 4. The TWS variations within the lake show much larger values and it is also more pronounced than other areas. This is expected due to the impact of surface water storage, which plays an important role in the lake.

### 4.3. Water Storage Trend

Despite the close spatial and temporal variations of different water components, their overall trend can be different as they show how much of this incremental precipitation is preserved within each water component. To study this, the correlation between the precipitation and TWS, surface water, and groundwater are calculated. Furthermore, groundwater, surface water, and TWS trends over the area for the study period are presented in Figure 9. The trend values are computed at each grid point using the corresponding time series. To statistically check the trend values, the Mann–Kendall Test [64] at 0.05 significance level is applied and the significant points are highlighted in the figure. A good pattern of agreement can be observed between the water storage components and precipitation over the period of 2019–2020. This is, however, more pronounced for TWS followed by surface water. Surface water and soil moisture included in TWS estimates largely follow the precipitation changes, hence the larger correlation achieved. Both surface water storage and TWS trends map show a positive increase in the lake area. This, to a lesser degree, can also be seen in the groundwater trend map. From the less significant groundwater trend in LV, it can be inferred that the large trend values in TWS are mostly caused by surface water changes (as also seen in Figure 4). Most of the trend values, particularly within the lake area are found to be significant, which can also be inferred from the significant trend maps. This shows the remarkable impact of recent precipitations on increased water storage components in the region. Over the northern part of the lake, a larger positive groundwater trend can be observed mostly in groundwater and TWS maps, thus, groundwater can be the main reason behind the TWS positive trends.

### 4.4. Principal Component Analysis (PCA)

The PCA applied here can help to better detect the temporal variations in the time series. Figure 10 demonstrates the PCA results and corresponding Empirical Orthogonal Function (EOF) for the TWS changes and Precipitation for the first three extracted dominant modes. The major trends can be observed in PC1, where both TWS and precipitation show a similar pattern between January 2019 and January 2020. A strong positive anomaly is characterised in April 2019 in both time series. The second considerable positive trend occurred more gradually from July 2019, which is caused by consistent rainfall over the same period. These anomalies can be seen from EOF1 to have large impacts in southern parts of the Nile Basins mostly affecting the Lake Victoria region. Greater positive TWS and precipitation can be observed in the southern parts of the lake. Two noticeable anomalies in February to early March 2019 can be found in PC2 TWS time series that match a positive and negative anomaly in PC2 precipitation time series. The two anomalies are responsible for the water storage increases in the northern parts of the lake (cf. EOF2). This is also extended to the eastern side of the Nile basin. Both time series also show another sharp anomaly in early April 2019. These fast TWS change events are majorly induced by short-term precipitations. Further, high magnitude variations for PC3 precipitation and TWS change time series exist for the period of July 2019 to December 2019 (as also reflected in PC1) affecting mostly the south-eastern part of Lake Victoria (EOF3). This event is followed by a considerable decline in January 2020. From PC3 precipitation, it can be seen that large variations begin to disappear from January 2020 leading to the negative TWS change trend (with ∼2 months lag) from April 2020 after the next major rainfall event. This has a large influence on the lake’s western side where negative TWS can be found.

### 4.5. Climate Impacts

**Figure 11:**
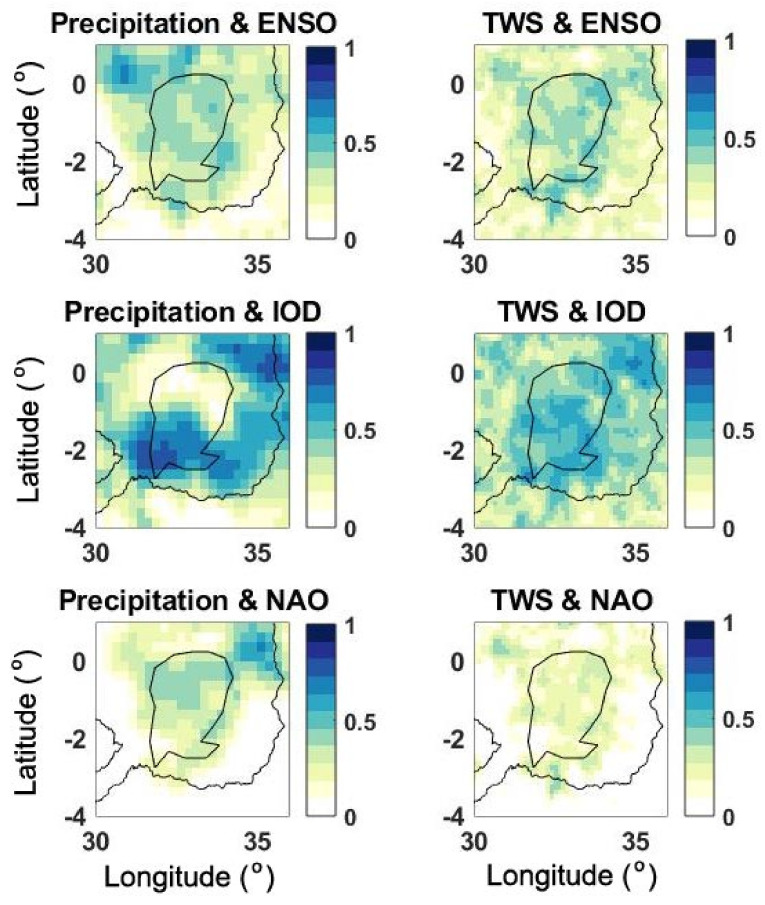
Correlation maps calculated between three climate indicators (top: ENSO, middle: IOD, bottom: NAO) and precipitation (**left**) as well as TWS (**right**).

**Figure 12:**
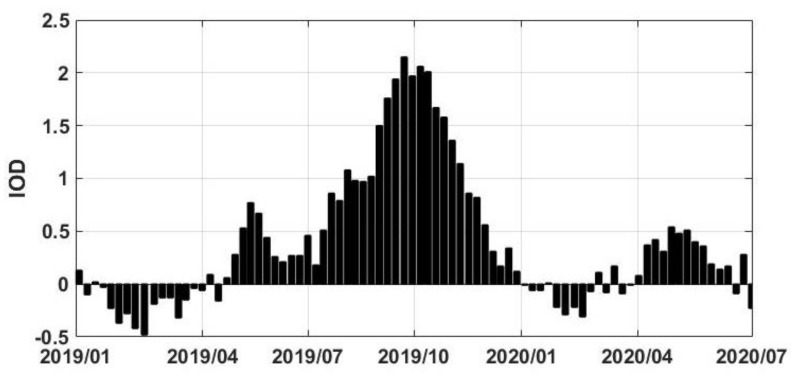
IOD time series for the study period.

The substantial impact of recent precipitation on various water storage components was observed in the preceding sections. In this section, we explore the connection between major climate indicators and these changes. Previous studies have indicated the historical impact of climate variabilities (such as IOD) on the region’s rainfall and water changes [12,34]. Such analysis can help to identify the potential reasons for higher than usual precipitation that occurred from 2019 to 2020. To analyse this impact on LV’s recent changes, ENSO, IOD, and NAO indices are used. We calculate the correlation between precipitation and TWS change time series at each grid point separately with the three indices (Figure 11). It is clear that IOD has the highest correlation values to precipitation and TWS changes that indicate the larger impact of IOD in the region’s climate and corresponding changes in the water storages during the period 2019–2020. It is found that ENSO and NAO have no significant impact by average correlation values of 0.32 and 0.27, respectively, within LV. A large positive IOD in 2019 (Figure 12) has a major impact on the LV region’s recent precipitation raise. This strong anomaly, which reached the level of the strongest events that occurred in 1994 and 1997, caused disasters in several countries around the Indian Ocean [65]. Large correlation between IOD and precipitation in the LV region is, therefore, suggests that the primary cause of LV’s water storage rise in 2019–2020 period as well as water level increase was IOD.

## 5. Discussion

Major precipitation event over the 2019–2020 period affected the south-eastern parts of Africa, and particularly Lake Victoria. The above-average precipitation rate for this period, i.e., ∼11% more for 2019–2020 compared to the 2002–2018 period, is found to be largely due to the IOD impact with an average correlation of 0.69. The phenomena results in significant water storage increase over the lake’s region with a positive trends across the region for various water compartments. The results obtained by merging satellite data and model simulations, are found to enhance the model performance compared to the altimetry and GRACE products. The correlation results indicate 0.23 improvement for surface water, and 0.18 for TWS estimates with respect to the simulations without data assimilation. These agree with previous findings of data assimilation studies. Ref. [21] reported 33% improvement in water estimates by assimilating altimetry-derived products. Multiple studies have also obtained TWS improvements when assimilating GRACE TWS products (e.g., [29,30,31,48,49]).

The 2019–2020 climatic event caused the highest increase water stored within the region (approximately) over the past 20 years. The TWS trend is more pronounced compared to groundwater and to a lesser degree surface storage due to (1) inclusion of multiple components such as soil moisture, surface and groundwater, and (2) its faster response to precipitation change. There is, still, a two-month delay for TWS to reach maximum correlation to the precipitation, which can be explained by the groundwater effects (e.g., [66]), as well as inflow water discharge from upstream rivers such as Kagera, Gurumeti, and Simiyu. The increased water storage also affects the TWS seasonality for the years 2019 and 2020. Higher water storage than normal (i.e., the period of 2002 to 2018) is observed for both wet (September to November) and dry (June to August) periods. This is, however, clearer for December to January leading to record-high water storage in the area compared to the previous years.

## 6. Conclusions

The significance of Lake Victoria as a primary source of fresh water for many East African residents makes the constant monitoring of its storage essential for better management. This study presents an innovative approach relying on models and space measurements to study the 2019–2020 lake’s water storage changes. The results show that:

(i) Recent intense precipitations particularly during 2019 caused an enormous water storage increase over the lake (∼25% TWS increase with respect to the period 2002–2018). Based on altimetry data, Lake Victoria’s water level rose by ∼1.4 m. This, according to the estimated water storage from data assimilation led to approximately 1750 gigatonne volume increase. A positive trend is observed in the past two years in the lakes water storage increase with two major events in April–May 2019 and December 2019–January 2020. Increased precipitation is found to be the main factor for these large positive water storage anomalies.
(ii) The overall trend, however, begins a negative pattern from early 2020 as seen by all the time series including TWS change and water level data. The impact of large scale climate variabilities on water changes within the area is also investigated using climate indicators including ENSO, IOD, NAO. Amongst these, IOD is found to be the main player that caused the above-average precipitation due to a strong positive IOD phenomenon mostly in the second half of 2019.

## Figures and Tables

**Table 1:** List of the datasets used for the study.

Data	Index	Reference
Hydrologic model	W3RA	CSIRO
Forcing sets	ERA-5	[47]
Terrestrial water storage (TWS)	GRACE-FO	[37]
Water level	Jason-3	NODC
Precipitation	GPM	GES DISC
Ocean-atmosphere indices	ENSO-IOD-NAO	NOAA-CPC

## Data Availability

Not applicable.
